# West Nile Virus Monitoring in Flanders (Belgium) During 2022–2023 Reveals Endemic Usutu Virus Circulation in Birds

**DOI:** 10.1155/tbed/4146156

**Published:** 2024-12-13

**Authors:** C. Sohier, F. C. Breman, M. Vervaeke, N. De Regge

**Affiliations:** ^1^Unit Exotic and Vector Borne Diseases, Sciensano 1180, Ukkel, Belgium; ^2^Agency for Nature and Forests, Brussels 1000, Belgium

## Abstract

The recent emergence of West Nile virus (WNV) and Usutu virus (USUV) in some European countries has triggered an increase in animal and human cases across Europe. Wild birds, serving as key reservoirs for WNV and USUV, often act as crucial indicators for the introduction and spread of these viruses. Currently, there is no durable large-scale monitoring for WNV in Belgium, and specific monitoring for USUV is lacking. In Flanders, passive WNV monitoring in birds has been in place for many years, while initial efforts to initiate active monitoring started in 2022. Here, we present the results of a limited study conducted during the vector seasons of 2022 and 2023 in Flemish bird populations to actively and passively monitor the prevalence of WNV and additionally assess the presence of USUV. Several real-time reverse transcription-PCR tests were employed for virus detection, revealing the absence of WNV-RNA during both vector seasons. Conversely, USUV-RNA was identified in 2022 through active surveillance, affecting two (5.5%) out of 36 birds (*Corvus corone*), and in passive surveillance, impacting eight (72.7%) out of 11 birds (*Turdus merula* [6] and *Rhea pennata* [2]). In 2023, active surveillance was more extensive and identified 16 (7.2%) USUV-RNA positive birds (*Buteo buteo* [1], *T. merula* [14] and *Athene noctua* [1]) out of 222 examined birds, while passive surveillance detected two (7.1%) positive birds (*T. merula* [1], and *Larus marinus* [1]) out of 28. Viral sequence information was obtained from seven USUV-positive birds using whole genome sequencing or Sanger sequencing. Phylogenetic analysis placed all identified strains within the Africa 3 lineage. This restricted WVN monitoring effort in Flanders did not reveal WNV presence, but found indications of an endemic USUV circulation in Belgium. It is crucial to intensify monitoring efforts for WNV in the coming years, considering its endemic status in several European countries and its expanding geographical range in northern Europe.

## 1. Introduction

West Nile virus (WNV) and Usutu virus (USUV) belong to the *Flavivirus* genus in the *Flaviviridae* family [1, 2]. Both viruses possess a single-stranded positive-sense RNA genome enclosed in spherical and enveloped virions [1, 3]. In their natural cycle, WNV and USUV primarily utilize *Culex* mosquitoes as their main vector, with various bird species acting as reservoirs for the virus.

First isolated in Uganda in 1937, WNV has since then been identified globally, with phylogenetic analyses revealing nine distinct evolutionary lineages. Lineage 1 WNV strains have circulated in Europe and the Mediterranean Basin since at least the late 1950s, causing sporadic infections and outbreaks in humans and animals [4]. In 2004, a lineage 2 WNV strain emerged in central Europe, leading to outbreaks of neuroinvasive disease in humans and animals, spreading to several central and southern European countries. Another lineage 2 strain surfaced in eastern Europe in 2007, subsequently spreading to southern Europe [5, 6]. In recent years, 17 European countries, including France and the Netherlands, reported cases of WNV in animals or humans [7]. Thus far, no indigenous infections with WNV infections have been detected in Belgium; only seven introduced cases of WNV in humans were reported between 2012 and 2022 in Belgium [8]. Despite this absence, the risk of its introduction is notable due to the close geographical proximity to affected regions and the presence of vectors and hosts. There remains the possibility that the virus is already present but remains undiagnosed due to limited WNV monitoring efforts. Only restricted passive monitoring efforts for WNV in birds were conducted in Flanders, Belgium, from the end of 2016 onward [9], with no reported cases of diagnosed WNV in birds.

USUV was, historically, limited to Africa, where it is not known to cause bird mortalities. USUV made its European debut in Italy in 1996, followed by outbreaks in Austria in 2001 that resulted in numerous dead Eurasian blackbirds (*Turdus merula*) [10]. Since then, USUV circulation has been detected in many western, southern, and central European countries, predominantly in birds and mosquitoes, but also in various mammalian species such as rodents (shrews) [11], dogs [12], bats [13], red deer [14], wild boar [15], equids [16], and humans [17]. USUV isolates are currently classified into eight lineages (Africa 1–3 and Europe 1–5) [18]. In Belgium, USUV infection was initially detected in the Meuse Valley in 2012 in a captive Eurasian bullfinch (*Pyrrhula pyrrhula*) and a wild, great spotted woodpecker (*Dendrocopos major*) [19]. Subsequently, in 2016, widespread bird mortalities linked to USUV infection were reported across the country [9, 18, 20, 21]. In 2017 and 2018, USUV-RNA was also detected in birds and bats in Belgium [22]. Surveillance based on partial gene sequencing, specifically the nonstructural protein 5 (NS5) gene, revealed that most Belgian strains belonged to the Europe 3 lineage, with some belonging to the Africa 3 lineage. One USUV strain manifested a close genetic relationship with the European 1 strains [23]. No data regarding the detection of USUV have been published from 2018 until now. Similar to WNV, only limited passive monitoring efforts for USUV in birds were conducted in Flanders, Belgium, from the end of 2016 onwards. Nevertheless, the identification of USUV-specific antibodies in the sera of Belgian wild boars in 2019 and 2020 [15] provides potential evidence indicating the endemic presence of USUV in Belgium.

Given the increasing circulation of WNV in Europe, there is a growing demand for more intensive WNV monitoring in Belgium. Wild birds play a crucial role as reservoirs and can serve as key indicators in monitoring their introduction and spread. Therefore, to gather up-to-date information on virus activity in Belgium and gain insights into the phylogenetic relationships among circulating strains, passive as well as active surveillance was conducted in 2022 and 2023 in birds. This surveillance specifically targeted WNV, but due to the implemented test cascade, additional testing for USUV was also carried out.

## 2. Method

### 2.1. Passive Monitoring

Since 2016, a passive surveillance program was established in Flanders primarily aiming at detecting WNV circulation but the program has also been used to monitor USUV. Since then, wildlife rescue centers, veterinarians, and the Antwerp Zoo have been requested to freeze and submit deceased bird species susceptible to either WNV or USUV or to collect swabs from them. Samples collected included brain, heart, lungs, throat, and cloacal swabs. This paper specifically discusses samples collected in 2022 and 2023. All samples were stored at −20°C until transportation at 4°C to Sciensano, where they were analyzed.

### 2.2. Active Monitoring

Since 2022, the first efforts have been made to start also an active WNV monitoring in wild birds in Flanders, using oropharyngeal swabs of birds submitted to a bird rescue center in Oudsbergen (Province Limburg). All Corvidae and birds of prey (Accipitridae, Falconidae, Strigiformes) captured from July to the end of October underwent swabbing. Subsequently, during the 2023 season (May to the end of October), a broader spectrum of birds, including Corvidae, birds of prey (Accipitridae, Falconidae, Strigiformes), and songbirds (Passeriformes) were swabbed at both the bird rescue center in Oudsbergen (Province Limburg) and the bird rescue center in Brasschaat-Kapellen (Province Antwerp). The swabs were carefully placed in Amies medium (Copan) and stored at −20°C until transportation at 4°C, after which they were thoroughly analyzed.

### 2.3. RNA Extraction

#### 2.3.1. Swab Samples

Swab samples were homogenized by vortexing for 10 s. RNA extraction from the swab samples was performed using the QIAamp viral RNA kit (Qiagen, Antwerp, Belgium) following the manufacturer's protocol.

#### 2.3.2. Organ Samples

Organ samples (0.5 cm³) were transferred to a 2 mL Eppendorf tube containing beads and 1 mL of PBS and homogenized using a TissueLyser. The homogenization process consisted of two cycles of 2 min each at 25 Hz, followed by centrifugation at 10,000 rpm for 5 min. RNA extraction from the organ samples was conducted using the RNeasy Mini Kit (Qiagen, Antwerp, Belgium), according to the manufacturer's instructions. RNA was extracted from the supernatant of homogenized brain tissue or pooled organ samples (heart, lung, and brain).

### 2.4. WNV and USUV RNA Detection

To detect the presence of WNV or USUV, all RNA extracts underwent screening using four distinct real-time PCR assays. This was done to confirm the specific flavivirus, as cross-reactivity in PCR is a known phenomenon among these closely related flaviviruses [7]. The AgPath-ID one-step RT-PCR kit was used (Thermo Fisher) for amplification with following reaction mixture (12.5 μL RT-PCR Buffer [2x] AgPath-ID One-Step, 1 μL RT-PCR Enzyme Mix [25x] AgPath-ID One-Step, 2 μL Mix primer/probe, 4,5 μL Nuclease free water and 5 μL RNA sample). A final primer and probe concentration of 0.8 and 0.32 μM, respectively, was used for all PCR assays.

Two different sets of primers and probes, as described by Dridi et al. [24] and Linke et al. [25], were used to detect the presence of WNV RNA in the extracts. Similarly, for the detection of USUV RNA, two sets of primers and probes described by del Amo et al. [26] and Rouffaer et al. [9] were applied. In each run, negative extraction and negative and positive amplification controls were also included. Samples were run on a LightCycler480 according to the following temperature program: 45°C for 10 min and 95°C for 10 min, followed by 45 cycles at 95°C for 15 s and 60°C for 45 s. CT-values ≤ 40 and with curves that showed an exponential amplification were considered positive.

### 2.5. Virus Isolation

Virus isolation was attempted on organ samples from all six birds that tested positive for USUV by RT-PCR. For each sample, 100 μL of homogenized organ suspension was added to BHK21 cell monolayers cultured in 6-well plates. After 6 h of incubation at 37°C, the cells were washed with fresh DMEM containing 2% antibiotics. The medium was then replaced with fresh DMEM supplemented with 2% antibiotic-antimycotic and 10% fetal calf serum (FCS), followed by incubation at 37°C under 10% CO for 4 days. After incubation, the supernatant was collected and tested for viral presence using real-time PCR assays as previously described.

### 2.6. Whole Genome Sequencing

One sample from the viral isolation (one passage) was extracted using the Machery Nagel viral RNA/DNA extraction kit; it was then reverse transcribed using “MultiScribe Reverse Transcriptase” kit from ThermoFisher with the included random hexameric primers following the manufacturer's protocol. Double-stranded DNA was then created using the “NEBNext Ultra II Non-Directional RNA Second Strand Synthesis Module.” This was then purified using the magnetic bead extraction kit “Agencourt AMPure XP” from Beckman-Coulter following the manufacturer's protocol. Whole genome sequencing was done on the resulting DNA extract using Illumina MisSeq at the Sciensano Transversal activities in the Applied Genomics research group. Library preparation was done using the Nextera XT kit (Illumina). We generated paired-end reads of 250 bp length with an estimated insert size of 250 bp (MiSeq V3 chemistry). Resulting data were demultiplexed, and adapters and Illumina indices were removed before continuing with downstream analyses.

### 2.7. Sanger Sequencing of Partial USUV Sequences

All the samples that tested positive for USUV in RT-PCR (swabs and organs) were subjected to two traditional PCRs to obtain amplicons for sequencing: they were submitted to a Pan-flavivirus PCR targeting the NS5 gene with cFD2 and MAMD primers [27] and a PCR directed to amplify the gene E of USUV [28]. Afterward, the amplicons were Sanger sequenced, and the obtained sequences were aligned and inspected to detect mutations. The consensus sequences were then utilized for phylogenetic reconstruction.

### 2.8. Genome Mapping

The resulting data were mapped against the designated USUV reference (GenBank accession no: NC 006551.1). The genome was assembled and mapped on the Sciensano Galaxy server for genomics analyses. For read mapping, the BWA mapper was employed, and reads with a quality lower than 20 were removed. A first draft consensus sequence was generated using Samtools [29]. The total assembly was evaluated in the program “Tablet” [30]. The consensus was manually assessed and curated to verify polymorphisms and insertion-deletion (indel) sites.

### 2.9. WGS Data Used

All WGS for USUV that were available on 19-04-2023 were collected, in addition to two outgroup sequences for WNV strains 1 and 2 (GenBank accession numbers NC_009942 and NC_009943, respectively). Sequences were excluded when the sequence contained more >1% sequence ambiguities. The entire dataset was aligned using MAFFT online [31, 32] allowing for ambiguous sites to be included and subsequently manually evaluated and refined using MEGA11.0.3 [33]. The aligned dataset contained 380 whole genome sequences.

### 2.10. Phylogeny Reconstruction of the WGS-Based Sequence Dataset and the Dataset Based on USUV Surface Protein E Sequences

Phylogenetic analyses, used to visualize a hypothesis of evolutionary relationships of our datasets (both the WGS-based dataset and the dataset based on the sequences for prot E), were performed under maximum likelihood (ML) criteria using IQ-TREE multicore version 1.6.12 for Windows, run locally (iqtree.cibiv.univie.ac.at) [34–37]. Sequences that are exactly alike were discarded by IQ-TREE during the analysis and later added to the phylogeny to prevent the occurrence of inflated support values for nodes during bootstrapping. We used model selection (ModelFinder [23] to find the optimal model. Iqtree uses three criteria for model selection: (1) the Akaike information criterion (AIC), (2) the corrected AIC (cAIC), and (3) the Bayesian information criterion (BIC). In case of conflict, Iqtree employs the model selected by the BIC. However, all three selection criteria employed by Iqtree for model selection were in agreement, and for the WGS-based dataset, the GTR+F+R10 model was selected. We used bootstrapping (UFBoot) to generate 10,000 trees. We used the Shimodaira–Hasegawa-like approximate likelihood ratio test RT (SH-aLRT) [38], the approximate Bayes test, and bootstrapping to evaluate node support. We further employed nearest neighbor interchange (NNI) search to initialize the candidate set and increased this to 100 (as opposed to 20 under default settings), and during the likelihood search, we kept the 10 best trees during each step rather than the default of five. For a detailed overview of the models available and the selection, please see [39] and http://www.iqtree.org/doc/Substitution-Models) for more recent additions. Clades in the phylogeny are considered supported when the three employed methods of node evaluation, SH-aLRT [38], the approximate Bayes test (pp) and bootstrapping (BS-s) yielded statistical support of values >75/>97/75, respectively.

## 3. Results

### 3.1. Passive Monitoring

**Figure 1:**
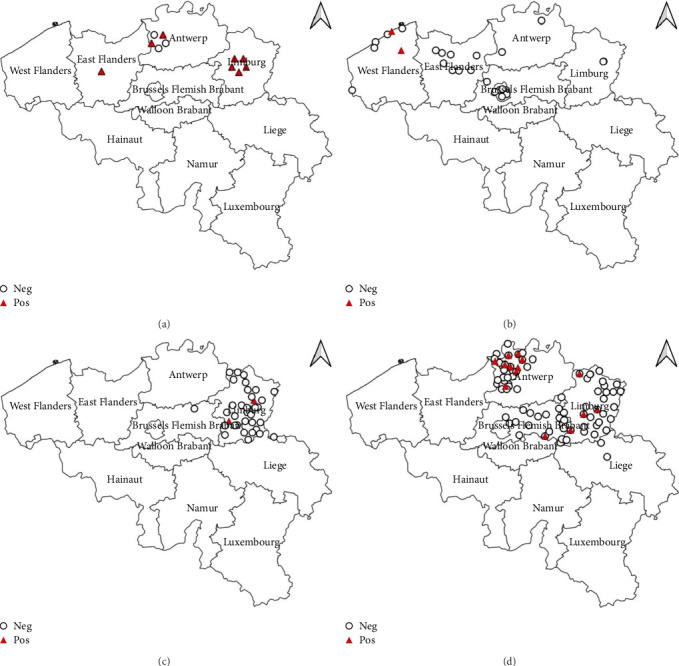
Positive and negative USUV RNA samples from (a) passive monitoring 2022, (b) passive monitoring 2023, (c) active monitoring 2022, and (d) active monitoring 2023. USUV, Usutu virus.

The passive monitoring effort in 2022 resulted in the sampling of a total of 11 birds (Table 1). These comprised six Eurasian Blackbirds (*T. merula*) submitted to bird rescue centers, as well as two African Penguins (*Spheniscus demersus*) and three Darwin's Rheas (*Rhea pennata*) submitted by the Zoo of Antwerp. None of these birds tested positive for WNV. However, eight out of the 11 sampled birds, specifically the six Eurasian Blackbirds (*T. merula*) and two Darwin's Rheas (*R. pennata*), tested positive for USUV. The cycle threshold (Ct) values ranged between 16 and 36. As can be seen in Figure 1, positive samples were identified in the provinces East-Flanders, Antwerp, and Limburg. Furthermore, USUV was successfully isolated from one of the USUV-positive Darwin's rheas.

In 2023, a total of 28 dead birds were sampled, all submitted to bird rescue centers (Table 1). Similar to the previous year, no positive samples for WNV were found. However, two birds (7.1%), specifically one Eurasian Blackbird (*T. merula*) and one Great Black-backed Gull (*Larus marinus*), tested positive for USUV with Ct values of 37.5 and 28, respectively. As can be seen in Figure 1, the two positive samples were located in the province West-Flanders.

### 3.2. Active Monitoring

The active monitoring effort resulted in a total of 36 birds sampled in 2022 from the bird rescue center in Oudsbergen and 222 birds in 2023 from the bird rescue centers in Oudsbergen and Brasschaat-Kapellen (Table 1). All samples collected in both 2022 and 2023 were negative for WNV, as detailed in Table 1. Notably, during the active monitoring in 2022, two out of the 36 birds tested positive for USUV through real-time PCR, constituting a 5.6% positivity rate (Figure 1). These positive cases exclusively involved crows (*Corvus corone*) with Ct's of 29.7 and 38.5. The 2023 active monitoring revealed 16 positive USUV birds (7.2%). The affected species were predominantly *T. merula* (14 cases) but also included one common buzzard (*Buteo buteo*) and one little owl (*Athene noctua*) (Figure 1) and were found in both sampled provinces (Antwerp and Limburg). The Ct's were ranging between 23 and 40.

### 3.3. Viral Isolation

After one passage on BHK21 cells, we successfully isolated the virus from only one of the six birds tested, specifically from a sample collected from Darwin's rhea.

### 3.4. USUV Whole Genome Sequencing

The WGS of the USUV strain present in one Darwin's rhea (*R. pennata*) from the passive monitoring of 2022 was sequenced. The resulting Illumina-based read library contained 332,918 reads associated with the USUV. This corresponds to 7% of the entire read library. The average read depth was 5574 reads. The total length of the reconstructed genome was 10,996 bp, which is in line with what is found for other USUVs but a bit shorter than the reference used (NC_006551.1 11,066 bp).

### 3.5. USUV-Based Sanger Sequencing

No amplicons were obtained with the Pan-flavivirus protocol targeting the NS5 gene. Nevertheless, seven amplicons of the USUV *E* gene were obtained and analyzed. These included five amplicons from swabs of European blackbirds (*T. merula*), one from a swab of a little owl (*A. noctua*), and one from brain tissue of a Darwin's rhea (*R. pennata*). The latter was also used for whole genome sequencing.

### 3.6. WGS-Based Phylogeny Reconstruction

**Figure 2:**
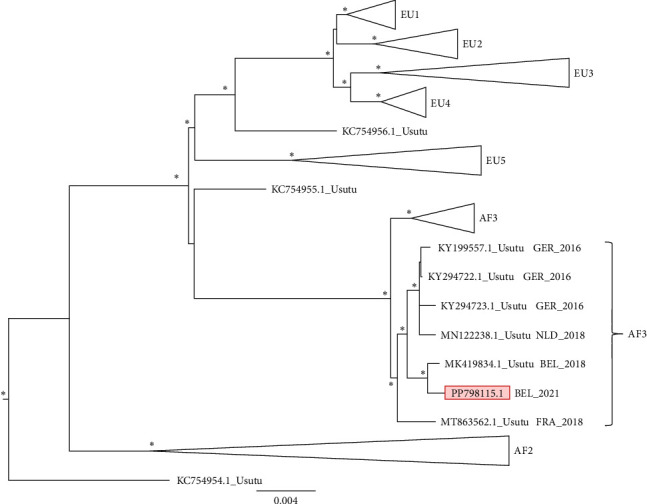
WGS-based phylogeny for USUV. Nodes (clades and subclades) supported by significant values (SH/pp/BS-s >75/>97/75) are marked with an *⁣*^*∗*^. The scale bar indicates substitutions per site. The outgroups (WNV1 and 2) have been removed from the figure to increase detail in the ingroup. The sequence generated during this study is indicated in red. USUV, Usutu virus.

The dataset contained 380 sequences, with maximum of 11,249 bp in length. There were 3691 parsimony-informative, 1462 singleton sites, and 6096 constant sites in the alignment. The sequence generated in this study was placed in the major clade “Africa 3^"^ and in a subclade with sequences from both Belgium and neighboring countries (Figure 2).

### 3.7. Gene E-Based Phylogeny Reconstruction

**Figure 3:**
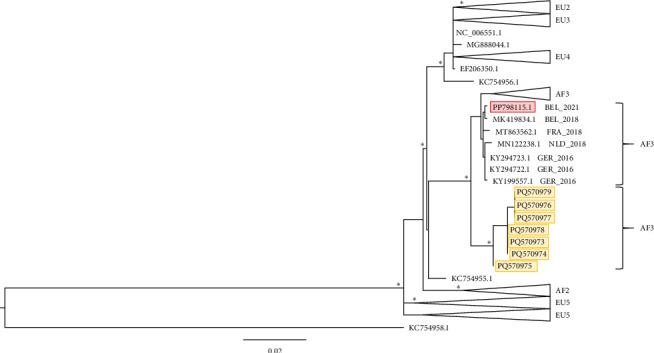
Phylogeny based on the USUV prot E sequence. Nodes (clades and subclades) supported by significant values (SH/pp/BS-s >75/>97/75) are marked with an *⁣*^*∗*^. The scale bar indicates substitutions per site. The outgroups (WNV1 and 2) have been removed from the figure to increase detail in the ingroup. The sequences generated during this study are indicated in orange. USUV, Usutu virus.

The dataset contained 387 sequences. The alignment length was 1506 bp, covering the entire exon encoding protein E. The length of the recovered partial sequences for the sequence of protein E ranged from 368 to 426 bp. There were 500 parsimony informative, 209 singleton sites, and 797 constant sites in the alignment. The sequences generated in this study were placed in the major clade “Africa 3.” They form a subclade which is sister to the clade with sequences from both Belgium and the neighboring countries (Figure 3).

## 4. Discussion

During our 2-year surveillance of the Belgian bird population, we did not detect WNV in any of the 297 samples collected. This is noteworthy, especially when considering the broader European map showing the spread of WNV (ECDC, EFSA, 2023). Given WNV's detection in neighboring countries (France, the Netherlands, and Germany) in recent years, it remains unclear whether our findings are influenced by the short viremic period and potential underdiagnosis due to limited monitoring. Notably, while WNV was detected in the Netherlands in 2020, it has not been reported there since, and no recent cases have been observed in northern France, suggesting that Belgium may currently be free from WNV. Nevertheless, it is essential to intensify monitoring efforts in the coming years, including conducting serological studies to more accurately assess WNV circulation in the region, especially given the current endemic WNV status in several EU/EEA countries and its expanding geographical range [9].

Monitoring birds is a valuable option as birds play an important role in virus transmission and the spread to new, previously unaffected areas [40, 41]. Furthermore, surveillance of WNV in mosquitoes and horses could prove useful in Belgium, serving as an additional early warning sign of WNV circulation. Particularly in endemic situations, monitoring mosquitoes and horses can be cost-effective, leading to earlier detection and enabling necessary actions such as targeted mosquito control, vaccination campaigns for horses, and public health awareness to prevent the further spread of the virus.

Due to the known cross-reactivity in PCR among various flaviviruses, often multiple PCR assays are conducted, especially when closely related flaviviruses, such as WNV and USUV, need to be distinguished [7]. In our surveillance efforts, we immediately implemented four PCR reactions, including USUV testing. This revealed the active circulation of USUV in Flanders, Belgium, aligning with previous findings detecting USUV in birds in 2018 [22] and the presence of USUV-specific antibodies in Belgian wild boar sera in 2019 and 2020 [15]. In our study, positive USUV samples were identified in all provinces of Flanders where sampling was conducted, indicating a geographic spread across the surveyed area. Among the USUV-positive birds, 75% were Eurasian blackbirds. This aligns with expectations, given the well-documented phenomenon that USUV can lead to a substantial die-off in Eurasian Blackbirds [7, 40, 42]. Therefore, particularly in passive surveillance involving deceased birds, the presence of positive USUV in Eurasian blackbirds can be anticipated, as was observed in Belgium in 2018 [9]. In our surveillance, we also noted a higher prevalence in passive monitoring compared to active monitoring in 2022, with 72.7% versus 5.5% of USUV-positive birds.

Furthermore, phylogenetic analysis revealed that our obtained sequences belong to the USUV Africa 3 lineage. This lineage, along with the Europe 1 and 3 lineages, has previously been identified in Belgium and other European countries [22, 43]. Overall, USUV appears to be endemic, with the circulation of one or possibly multiple strains of the virus. The endemicity is shown by its continued presence, spanning not only multiple years but also across various provinces in Flanders.

Our current limited sample size in Belgium allows us to maintain our testing cascade with four PCR assays to distinguish between WNV and USUV. In this context, we can prevent the misdiagnosis of USUV-positive samples as WNV infections. However, whenever the sample size increases, the associated costs also become high, leading to the need to revise our testing approach. We propose for large sample sizes to implement an initial pan-flavivirus PCR [44–46], followed by specific PCR assays on positive cases or the validation of positive samples through the sequencing of amplification products. An added advantage of using a pan-flavivirus PCR is its ability to detect a broad range of flaviviruses, which is valuable for surveillance, as flaviviruses are known to emerge unexpectedly in new regions.

In Belgium, as well as on the European level, the emphasis is on WNV, likely because it is a notifiable disease, unlike USUV. Initially, USUV was regarded as an arbovirus with low zoonotic potential. However, recent data from various European countries suggest the possibility of a significantly higher incidence of clinical neuroinvasive USUV infections in humans than previously assumed [47, 48]. The combination of this new understanding and the fact that USUV leads to substantial bird mortality underscores the importance of monitoring and tracking the spread of USUV.

## 5. Conclusions

Presently, there have been no detections of WNV in Belgian bird populations. Nonetheless, it is important to enhance surveillance efforts in the future, given the established presence of WNV in several European countries and its expanding range. Regarding the USUV, indications suggest it has become endemic in Belgium, with at least the circulation of lineage Africa 3 strains. Moreover, given the greater zoonotic potential of USUV than initially assumed, continued monitoring is crucial.

## Figures and Tables

**Table 1:** Number of birds tested positive or negative in the passive and active monitoring programs for WNV and USUV.

			WNV monitoring				USUV monitoring			
Order	Common name	Species name	Active 2022	Passive 2022	Active 2023	Passive 2023	Active 2022	Passive 2022	Active 2023	Passive 2023
Accipitriformes	Western marsh harrier	*Circus aeruginosus*	—	—	0/2	—	—	—	0/2	—
	Common buzzard	*B. buteo*	0/11	—	0/33	—	0/11	—	1/33	—
	Northern goshawk	*Accipiter gentilis*	0/1	—	0/5	—	0/1	—	0/5	—
	Eurasian sparrowhawk	*Accipiter nisus*	0/1	—	0/17	—	0/1	—	0/17	—
	European honey buzzard	*Pernis apivorus*	—	—	0/1	—	—	—	0/1	—

Charadriiformes	Great black-backed gull	*L. marinus*	—	—	—	0/1	—	—	—	1/1

Falconiformes	Eurasian hobby	*Falco subbuteo*	—	—	0/2	—	—	—	0/2	—
	Peregrine falcon	*Falco peregrinus*	0/2	—	0/5	—	0/2	—	0/5	—
	Merlin	*Falco columbarius*	—	—	0/2	—	—	—	0/2	—
	Common kestrel	*Falco tinnunculus*	0/2	—	0/15	—	0/2	—	0/15	—

Passeriformes	Eurasian magpie	*Pica pica*	0/2	—	0/14	0/3	0/2	—	0/14	0/3
	Eurasian jay	*Garrulus glandarius*	0/1	—	0/7	—	0/1	—	0/7	—
	European greenfinch	*Chloris chloris*	—	—	0/1	—	—	—	0/1	—
	House sparrow	*Passer domesticus*	—	—	0/5	—	—	—	0/5	—
	Western jackdaw	*Corvus monedula*	0/1	—	0/24	0/12	0/1	—	0/24	0/12
	Redwing	*Turdus iliacus*	—	—	0/1	—	—	—	0/1	—
	Carrion crow	*C. corone*	0/5	—	0/13	0/5	2/5	—	0/13	0/5
	Mistle thrush	*Turdus viscivorus*	—	—	0/1	—	—	—	0/1	—
	Eurasian blackbird	*T. merula*	—	0/6	0/33	0/1	—	6/6	14/33	1/1
	Rook	*Corvus frugilegus*	—	—	0/1	—	—	—	0/1	—
	European robin	*Erithacus rubecula*	—	—	0/2	—	—	—	0/2	—
	Common starling	*Sturnus vulgaris*	—	—	0/2	—	—	—	0/2	—
	Crow family	*Corvidae sp*.	—	—	—	0/5	—	—	—	0/5

Piciformes	Great spotted woodpecker	*D. major*	—	—	—	0/1	—	—	—	0/1

Rheiformes	Darwin's rhea	*R. pennata*	—	0/3	—	—	—	2/3	—	—

Sphenisciformes	African penguin	*S. demersus*	—	0/2	—	—	—	0/2	—	—

Strigiformes	Tawny owl	*Strix aluco*	—	—	0/10	—	—	—	0/10	—
	Barn owl	*Tyto alba*	0/4	—	0/14	—	0/4	—	0/14	—
	Eurasian eagle-owl	*Bubo bubo*	0/2	—	0/2	—	0/2	—	0/2	—
	Long-eared owl	*Asio otus*	0/1	—	0/4	—	0/1	—	0/4	—
	Little owl	*A. noctua*	0/3	—	0/6	—	0/3	—	1/6	—

Total positive	—	—	**0/36**	**0/11**	**0/222**	**0/28**	**2/36**	**8/11**	**16/222**	**2/28**

*Note*: The total positives are indicated in bold.

Abbreviations: USUV, Usutu virus; WNV, West Nile virus.

## Data Availability

The consensus sequence, coded PP798115, was submitted to GenBank. The partial sequences encoding USUV protein E have been submitted to GenBank with accession numbers PQ570973, PQ570974, PQ570975, PQ570976, PQ570977, PQ570978 and PQ570979.

## References

[1] Martín-Acebes M. A., Saiz J.-C. (2012). West Nile Virus: A Re-Emerging Pathogen Revisited. *World Journal of Virology*.

[2] Simmonds P., Becher P., Bukh J. (2017). ICTV Virus Taxonomy Profile: Flaviviridae. *Journal of General Virology*.

[3] Mukhopadhyay S., Kim B.-S., Chipman P. R., Rossmann M. G., Kuhn R. J. (2003). Structure of West Nile Virus. *Science*.

[4] Zeller H. G., Schuffenecker I. (2004). West Nile Virus: An Overview of Its Spread in Europe and the Mediterranean Basin in Contrast to Its Spread in the Americas. *European Journal of Clinical Microbiology & Infectious Diseases*.

[5] Hernández-Triana L. M., Jeffries C. L., Mansfield K. L., Carnell G., Fooks A. R., Johnson N. (2014). Emergence of West Nile Virus lineage 2 in Europe: A Review on the Introduction and Spread of a Mosquito-Borne Disease. *Frontiers in Public Health*.

[6] Rizzoli A., Jimenez-Clavero M. A., Barzon L. (2015). The Challenge of West Nile Virus in Europe: Knowledge Gaps and Research Priorities. *Eurosurveillance*.

[7] ECDC, EFSA (2023). Surveillance, Prevention and Control of West Nile Virus and Usutu Virus Infections in the EU/EEA. https://www.ecdc.europa.eu/en/publications-data/surveillance-prevention-and-control-west-nile-virus-and-usutu-virus-infections.

[8] Stefani G., Rebolledo J., Van Esbroeck M. (2022). Epidemiologische Surveillance Van Westnijle Koorts_Westnijle Virus (WNV). https://www.sciensano.be/sites/default/files/wnv_2022_nl.pdf.

[9] Rouffaer L. O., Steensels M., Verlinden M. (2018). Usutu Virus Epizootic and *Plasmodium* Coinfection in Eurasian Blackbirds (*Turdus merula*) in Flanders, Belgium. *Journal of Wildlife Diseases*.

[10] Weissenböck H., Bakonyi T., Rossi G., Mani P., Nowotny N. (2013). Usutu Virus, Italy, 1996. *Emerging Infectious Diseases*.

[11] Diagne M. M., Ndione M. H. D., Di Paola N. (2019). Usutu Virus Isolated from Rodents in Senegal. *Viruses*.

[12] Durand B., Haskouri H., Lowenski S., Vachiery N., Beck C., Lecollinet S. (2016). Seroprevalence of West Nile and Usutu Viruses in Military Working Horses and Dogs, Morocco, 2012: Dog as an Alternative WNV Sentinel Species? Epidemiol. *Epidemiology and Infection*.

[13] Cadar D., Becker N., Campos R. D. M., Börstler J., Jöst H., Schmidt-Chanasit J. (2014). Usutu Virus in Bats, Germany, 2013. *Emerging Infectious Diseases*.

[14] García-Bocanegra I., Paniagua J., Gutiérrez-Guzmán A. V. (2016). Spatio-Temporal Trends and Risk Factors Affecting West Nile Virus and Related Flavivirus Exposure in Spanish Wild Ruminants. *BMC Veterinary Research*.

[15] Trozzi G., Adjadj N. R., Vervaeke M., Matthijs S., Sohier C., De Regge N. (2023). Comparison of Serological Methods for Tick-Borne Encephalitis Virus-Specific Antibody Detection in Wild Boar and Sheep: Impact of the Screening Approach on the Estimated Seroprevalence. *Viruses*.

[16] Ben Hassine T., De Massis F., Calistri P. (2014). First Detection of Co-Circulation of West Nile and Usutu Viruses in Equids in the South-West of Tunisia. *Transboundary and Emerging Diseases*.

[17] Lupia T., Marletto F. P., Scuvera I. T. (2022). First Human Usutu Virus Reported in Asti (Piedmont, Italy, August 2022) and Early Follow-Up. *Tropical Medicine and Infectious Disease*.

[18] Cadar D., Lühken R., van der Jeugd H. (2017). Widespread Activity of Multiple Lineages of Usutu Virus, Western Europe, 2016. *Eurosurveillance*.

[19] Garigliany M.-M., Marlier D., Tenner-Racz K. (2014). Detection of Usutu Virus in a Bullfinch (*Pyrrhula pyrrhula*) and a Great Spotted Woodpecker (*Dendrocopos major*) in North-West Europe. *The Veterinary Journal*.

[20] Van Borm S., Lambrecht B., Vandenbussche F., Steensels M. (2017). Complete Coding Sequence of Usutu Virus Strain *Gracula religiosa*/U1609393/Belgium/2016 Obtained From the Brain Tissue of an Infected Captive Common Hill Myna (*Gracula religiosa*). *Genome Announcements*.

[21] Garigliany M., Linden A., Gilliau G. (2017). Usutu Virus, Belgium, 2016. *Infection, Genetics and Evolution*.

[22] Benzarti E., Sarlet M., Franssen M. (2020). Usutu Virus Epizootic in Belgium in 2017 and 2018: Evidence of Virus Endemization and Ongoing Introduction Events. *Vector-Borne and Zoonotic Diseases*.

[23] Kalyaanamoorthy S., Minh B. Q., Wong T. K. F., von Haeseler A., Jermiin L. S. (2017). ModelFinder: Fast Model Selection for Accurate Phylogenetic Estimates. *Nature Methods*.

[24] Dridi M., Van Den Berg T., Lecollinet S., Lambrecht B. (2015). Evaluation of the Pathogenicity of West Nile Virus (WNV) lineage 2 Strains in a SPF Chicken Model of Infection: NS3-249Pro Mutation Is Neither Sufficient nor Necessary for Conferring Virulence. *Veterinary Research*.

[25] Linke S., Ellerbrok H., Niedrig M., Nitsche A., Pauli G. (2007). Detection of West Nile Virus Lineages 1 and 2 by Real-Time PCR. *Journal of Virological Methods*.

[26] Del Amo J., Sotelo E., Fernández-Pinero J. (2013). A Novel Quantitative Multiplex Real-Time RT-PCR for the Simultaneous Detection and Differentiation of West Nile Virus Lineages 1 and 2, and of Usutu Virus. *Journal of Virological Methods*.

[27] Scaramozzino N., Crance J.-M., Jouan A., DeBriel D. A., Stoll F., Garin D. (2001). Comparison of Flavivirus Universal Primer Pairs and Development of a Rapid, Highly Sensitive Heminested Reverse Transcription-PCR Assay for Detection of Flaviviruses Targeted to a Conserved Region of the NS5 Gene Sequences. *Journal of Clinical Microbiology*.

[28] Manarolla G., Bakonyi T., Gallazzi D. (2010). Usutu Virus in Wild Birds in Northern Italy. *Veterinary Microbiology*.

[29] Danecek P., Bonfield J. K., Liddle J. (2021). Twelve Years of SAMtools and BCFtools. *GigaScience*.

[30] Milne I., Stephen G., Bayer M. (2013). Using Tablet for Visual Exploration of Second-Generation Sequencing Data. *Briefings in Bioinformatics*.

[31] Kuraku S., Zmasek C. M., Nishimura O., Katoh K. (2013). ALeaves Facilitates on-Demand Exploration of Metazoan Gene Family Trees on MAFFT Sequence Alignment Server with Enhanced Interactivity. *Nucleic Acids Research*.

[32] Katoh K., Rozewicki J., Yamada K. D. (2019). MAFFT Online Service: Multiple Sequence Alignment, Interactive Sequence Choice and Visualization. *Briefings in Bioinformatics*.

[33] Tamura K., Stecher G., Kumar S. (2021). MEGA11: Molecular Evolutionary Genetics Analysis Version 11. *Molecular Biology and Evolution*.

[34] Nguyen L.-T., Schmidt H. A., von Haeseler A., Minh B. Q. (2015). IQ-TREE: A Fast and Effective Stochastic Algorithm for Estimating Maximum-Likelihood Phylogenies. *Molecular Biology and Evolution*.

[35] Trifinopoulos J., Nguyen L.-T., von Haeseler A., Minh B. Q. (2016). W-IQ-TREE: A Fast Online Phylogenetic Tool for Maximum Likelihood Analysis. *Nucleic Acids Research*.

[36] Hoang D. T., Chernomor O., von Haeseler A., Minh B. Q., Vinh L. S. (2018). UFBoot2: Improving the Ultrafast Bootstrap Approximation. *Molecular Biology and Evolution*.

[37] Breman F. C., Haegeman A., Krešić N., Philips W., De Regge N. (2023). Lumpy Skin Disease Virus Genome Sequence Analysis: Putative Spatio-Temporal Epidemiology, Single Gene Versus Whole Genome Phylogeny and Genomic Evolution. *Viruses*.

[38] Guindon S., Dufayard J.-F., Lefort V., Anisimova M., Hordijk W., Gascuel O. (2010). New Algorithms and Methods to Estimate Maximum-Likelihood Phylogenies: Assessing the Performance of PhyML 3.0. *Systematic Biology*.

[39] Posada D., Crandall K. A. (1998). MODELTEST: Testing the Model of DNA Substitution. *Bioinformatics*.

[40] Michel F., Fischer D., Eiden M. (2018). West Nile Virus and Usutu Virus Monitoring of Wild Birds in Germany. *International Journal of Environmental Research and Public Health*.

[41] Ziegler U., Bergmann F., Fischer D. (2022). Spread of West Nile Virus and Usutu Virus in the German Bird Population, 2019–2020. *Microorganisms*.

[42] Rijks J., Kik M., Slaterus R. (2016). Widespread Usutu Virus Outbreak in Birds in the Netherlands, 2016. *Eurosurveillance*.

[43] Siljic M., Sehovic R., Jankovic M. (2023). Evolutionary Dynamics of Usutu Virus: Worldwide Dispersal Patterns and Transmission Dynamics in Europe. *Frontiers in Microbiology*.

[44] Moureau G., Temmam S., Gonzalez J. P., Charrel R. N., Grard G., de Lamballerie X. (2007). A Real-Time RT-PCR Method for the Universal Detection and Identification of Flaviviruses. *Vector-Borne and Zoonotic Diseases*.

[45] Johnson N., Wakeley P. R., Mansfield K. L. (2010). Assessment of a Novel Real-Time Pan-Flavivirus RT-Polymerase Chain Reaction. *Vector-Borne and Zoonotic Diseases*.

[46] Vina-Rodriguez A., Sachse K., Ziegler U. (2017). A Novel Pan-*Flavivirus* Detection and Identification Assay Based on RT-qPCR and Microarray. *BioMed Research International*.

[47] Clé M., Beck C., Salinas S. (2019). Usutu Virus: A New Threat?. *Epidemiology and Infection*.

[48] Cadar D., Simonin Y. (2023). Human Usutu Virus Infections in Europe: A New Risk on Horizon?. *Viruses*.

